# Hierarchical Ta-Doped TiO_2_ Nanorod Arrays with Improved Charge Separation for Photoelectrochemical Water Oxidation under FTO Side Illumination

**DOI:** 10.3390/nano8120983

**Published:** 2018-11-28

**Authors:** Shiman He, Yuying Meng, Yangfei Cao, Senchuan Huang, Jingling Yang, Shengfu Tong, Mingmei Wu

**Affiliations:** School of Chemistry, Sun Yat-Sen University, No. 135, Xingang Xi Road, Guangzhou 510275, China; heshm3@mail2.sysu.edu.cn (S.H.); caoyf8@mail2.sysu.edu.cn (Y.C.); huangsch3@mail2.sysu.edu.cn (S.H.); yatouguang1@gmail.com (J.Y.); tongshf@mail.sysu.edu.cn (S.T.)

**Keywords:** hierarchical TiO_2_, Ta doping, charge separation, photoelectrochemical water oxidation

## Abstract

TiO_2_ is one of the most attractive semiconductors for use as a photoanode for photoelectrochemical (PEC) water oxidation. However, the large-scale application of TiO_2_ photoanodes is restricted due to a short hole diffusion length and low electron mobility, which can be addressed by metal doping and surface decorating. In this paper we report the successful synthesis of hierarchical Ta doped TiO_2_ nanorod arrays, with nanoparticles on the top (Ta:TiO_2_), on F-doped tin oxide (FTO) glass by a hydrothermal method, and its application as photoanodes for photoelectrochemical water oxidation. It has been found that the incorporation of Ta^5+^ in the TiO_2_ lattice can decrease the diameter of surface TiO_2_ nanoparticles. Ta:TiO_2_-140, obtained with a moderate Ta concentration, yields a photocurrent of ∼1.36 mA cm^−2^ at 1.23 V vs. a reversible hydrogen electrode (RHE) under FTO side illumination. The large photocurrent is attributed to the large interface area of the surface TiO_2_ nanoparticles and the good electron conductivity due to Ta doping. Besides, the electron trap-free model illustrates that Ta:TiO_2_ affords higher transport speed and lower electron resistance when under FTO side illumination.

## 1. Introduction

With the growing demand for energy and the concern of environmental problems worldwide, solar energy has long been regarded as one of the cleanest and renewable energy sources to address these challenges [1]. From an unlimited solar energy perspective, it is highly desirable to convert sunlight into an energy storage medium to provide continuous and stable power. Among sustainable energy systems, photoelectrochemical (PEC) water splitting is an attractive, clean, and environmentally-friendly approach to producing oxygen and hydrogen using sunlight and bias voltage [2]. A key target in PEC research continues to be the development of novel and efficient semiconductor photoelectrocatalysts. TiO_2_ has been one of the most promising candidates among various semiconductors since the pioneering work demonstrated by Fujishima and Honda in 1972, because of its chemical stability, cost effectiveness, and nontoxicity [3]. However, TiO_2_ still suffers from several challenges for large-scale applications, such as it’s short minority carrier (hole) diffusion length (10–100 nm) and low electron mobility (1 cm^2^ V^−1^ S^−1^). Thus, many efforts have been devoted to addressing these issues [4].

Recent work has been focused on one-dimensional (1-D) nanostructures such as nanotubes, nanowires, and nanorods, since they can improve the charge separation, charge transfer, and light absorption of photoelectrodes [5,6]. The 1-D TiO_2_ nanostructure could efficiently transport the charges between the TiO_2_ photoelectrode and electrolyte along the axial direction of the nanostructures [7]. However, comparing with nanoparticles, 1-D nanostructures have relatively smaller surface areas, which has a negative impact on the charge transfer process, especially in the case of sluggish anodic water oxidation [8]. Therefore, it is highly desirable to combine the advantage of surface nanoparticles (more surface active sites) and 1-D structures (highly-efficient charge transport along the axial direction) to enhance the overall PEC activity. In addition, doping TiO_2_ with other metal elements, such as tantalum, has been widely investigated, as it can tailor the band structure and increase the electric conductivity of TiO_2_ [9,10]. Until now, most of the Ta doping TiO_2_ nanomaterials have been explored for photocatalysis and dye-sensitized solar cells, while only a few works reported them as photoanodes in PEC water oxidation. Besides, the afforded photocurrents in water splitting have remained quite low over the past few years [10,11]. Typically, TiO_2_ PEC cells tested under illumination incident from the F-doped tin oxide (FTO) side (FS illumination) achieve larger current density than those under illumination incident from the TiO_2_ side (TS illumination) [12]. However, there is no report on employing the charge injection and separation efficiency to analyze the improved performance for Ta doped TiO_2_ photoanodes under FS illumination. Therefore, we believe that investigation of the mechanism of charge injection and separation inside TiO_2_ nanostructures under different light illumination models will help us find ways to improve the PEC efficiency of TiO_2_-based cells.

In this work, hierarchical Ta doped TiO_2_ nanorod arrays, with nanoparticles on the top, on FTO glass (Ta:TiO_2_), were synthesized by the hydrothermal method. Ta precursor plays an important role in the formation of the nanoparticles on the top of nanorod arrays. We demonstrate that the efficiency of the TiO_2_ nanorod arrays can be improved by surface nanoparticles and Ta doping. Moreover, these materials give a higher photocurrent under FTO illumination than those under TiO_2_ illumination, because of the faster electron diffusion and lower charge recombination.

## 2. Materials and Methods

### 2.1. Chemicals and Reagents

Hydrochloric acid (HCl 37.5% *wt*./*wt*.), acetone, absolute ethanol, and *n*-hexane were purchased from Guangdong Guanghua Sci-Tech Co., Ltd. (Guangzhou, China). Tetrabutyl titanate (≥99.0%), monopotassium phosphate, dipotassium phosphate, and potassium hydroxide were acquired from the Aladdin Industrial Corporation (Shanghai, China). Sodium sulfite was purchased from the Guangzhou Chemical Reagent Factory (Guangzhou, China). Ta(V) isopropoxide (99.9%, 10% *w*/*v* in isopropyl alcohol/*n*-hexane) was acquired from Alfa Aesar Co., Ltd. (Waltham, MA, USA). Fluorine-doped tin oxide glass (FTO, 2.2 mm thick, 7 Ω cm^−2^, Pilkington) was obtained from the Solar Energy Technology Co., Ltd., Wuhan, China. Deionized water was used throughout the experiment.

### 2.2. Synthesis of Hierarchical Ta-Doped TiO_2_ Nanorod Arrays with Nanoparticles on the Top, on FTO Glass (Ta:TiO_2_)

FTO glass was firstly cut into small pieces with a size of 1.5 cm × 2.0 cm, followed by treatment with sonication in acetone, water, and ethanol for 30 min each step. In a typical synthetic process, 140 μL of Ta isopropoxide (TIO) was dropwise added into an autoclave (15 mL) containing 7 mL of *n*-hexane, 0.7 mL of hydrochloric acid, and 0.7 mL of tetrabutyl titanate (TBT). Then, a piece of FTO substrate was placed into the above autoclave with an angle of 30° against the wall of the Teflon-liner, after which the autoclave underwent solvothermal treatment for 10 h at 180 °C. Once it was cooled to room temperature naturally, the samples were collected and thoroughly rinsed with ethanol and water, separately. After being dried under ambient conditions, the material was annealed in a furnace at 550 °C for 2 h under air atmosphere. Moreover, various materials, denoted as Ta:TiO_2_-*v*, where *v* (0–340 µL) is the volume of Ta isopropoxide (TIO), were obtained to investigate the effect of the TIO amount on PEC activity. In addition, Ta-doped TiO_2_ arrays with different lengths (or thickness of the Ta-doped TiO_2_ layer) on the substrate were prepared by a similar procedure, with different amounts of TBT (0.3 to 0.8 mL).

The crystalline structure of the materials was measured on an X-ray diffractometer (Rigaku D/max, Tokyo, Japan) operating with a Cu *Kα* source (*λ* = 1.5406 Å) in a 2*θ* range from 10° to 80° at a scan rate of 10° min^−1^. X-ray photoelectron spectroscopy (XPS) measurement was performed on a Thermo VG Scientific ESCALAB 250 spectrometer (Waltham, MA, USA) with a monochromatic X-ray source (Al *Kα*). The morphology was verified using a field-emission scanning electron microscope (FESEM, FEI Quanta 400, Hillsboro, OR, USA) operated at 20 kV. A FEI Tecnai G2 F30 transmission electron microscope (Hillsboro, OR, USA) equipped with a high angle annular dark field (HAADF) detector operated at 300 kV was used to obtain the transmission electron microscopy (TEM) images. A Shimadzu UV-3600 spectrophotometer (Kyoto, Japan) equipped with an integrating sphere attachment was chosen to record ultraviolet-visible (UV-Vis) diffuse reflectance spectra using BaSO_4_ as the reference.

Photoelectrochemical characterization of Ta-doped TiO_2_ photoanodes was carried out on an electrochemical workstation (Metrohm AutoLab PGSTAT302N, Herisau, Switzerland) with a home-built three-electrode optical cell, where the obtained Ta-doped TiO_2_ materials, Ag/AgCl, and Pt wire served as working, reference, and counter electrodes, respectively. The linear sweep voltammogram (LSV) curves over different materials were conducted with a scan rate of 20 mV s^−1^ in 1.0 M KOH aqueous solution (pH = 13.6). The illumination source was a 300 W Xe lamp (Perfect light PLS-SXE300CUV, Beijing, China) with an AM 1.5 global filter, calibrated with a standard Si solar cell to simulate AM 1.5 G illumination (100 mW cm^−2^). Potentials versus (vs.) the reversible hydrogen electrode (RHE) were calculated from the potentials vs. Ag/AgCl using the Nernst equation—*E*_RHE_ = *E*_Ag/AgCl_ + 0.0591(pH) + 0.196. Incident-photon-to-current-conversion efficiency (IPCE) was obtained using a solar simulator (Newport 69911 300 W xenon lamp, Irvine, CA, USA) coupled with an aligned monochromator (Oriel Cornerstone 260 1/4 m, Irvine, CA, USA). The electrochemical impedance spectroscopy (EIS) measurement was performed under the open-circuit condition, with 10 mV amplitude in a frequency range from 0.1 to 10 kHz with or without illumination. The donor density was acquired by Mott–Schottky measurement at 1 kHz.

## 3. Results and Discussion

### 3.1. Morphology and Structural Characteristics

**Figure 1:**
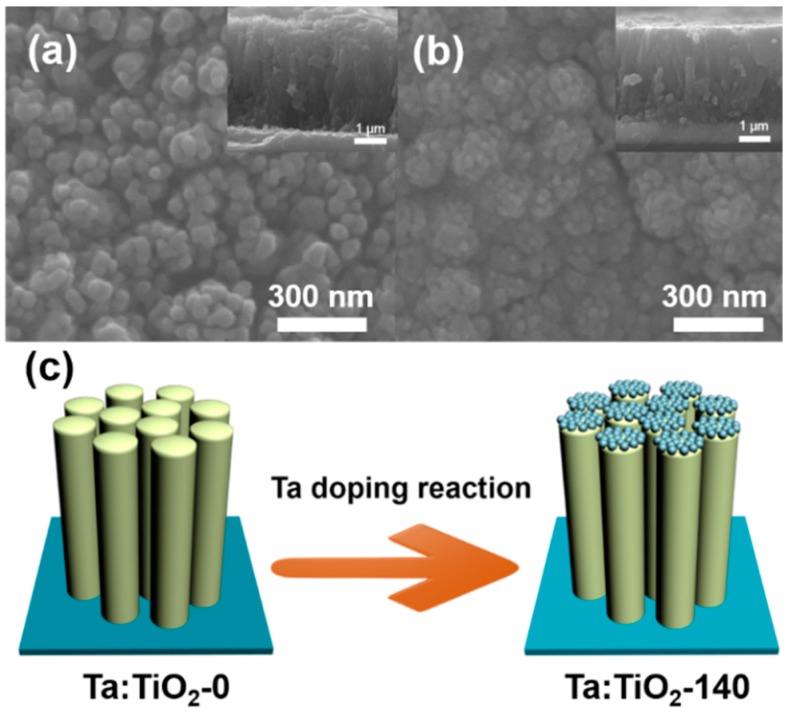
Surface SEM images of (**a**) Ta:TiO_2_-0 and (**b**) Ta:TiO_2_-140. The inserts in (**a**,**b**) are the corresponding cross images. (**c**) The schematic description of the morphology change after the Ta doping reaction.

Figure 1 shows the surface and cross SEM images of Ta:TiO_2_-0 and Ta:TiO_2_-140. The pristine TiO_2_ (Ta:TiO_2_-0) consisted of densely packed and vertically aligned nanorods with an average diameter of 150 nm and length of 3 μm. As shown in the cross image of Ta:TiO_2_-140 inserted in Figure 1b, there was no obvious difference in the nanorods’ diameter and length in comparison with Ta:TiO_2_-0, while viewed from the surface image, the diameter of the particles on the top of the nanorods was only 40 nm. Therefore, it is highly desirable to deeply investigate the structure of nanoparticles on the top of nanorods to understand the nanorods’ growth mechanism. In order to study the precursor’s influence on the Ta-doped TiO_2_ nanorods’ morphology, different amounts of Ta isopropoxide (TIO) were used. The surface and cross SEM images of variable Ta:TiO_2_-*v* nanorods are shown in Figures S1 and S2, respectively. Figure S1 shows that by increasing the TIO volume from 40 to 190 μL, the size of Ta:TiO_2_ nanoparticles on the top of nanorods gradually decreased from 150 to 40 nm. However, the TiO_2_ nanoparticles tended to be agglomerated on the nanorods’ surface when the TIO volume was further increased above 290 μL. Therefore, TIO must play an important role in the formation of the nanoparticles on the nanorod arrays (Figure 1c) [13]. On the contrary, the increase in TIO had no significant effect on the nanorods’ length, remaining consistent at 3 μm for all the Ta:TiO_2_-*v* materials. Moreover, the Ta:Ti (*at.*%) composition was investigated via SEM-based energy-dispersive X-ray spectroscopy (EDX), which shows that the Ta content increased with increasing TIO content (Table S1).

**Figure 2:**
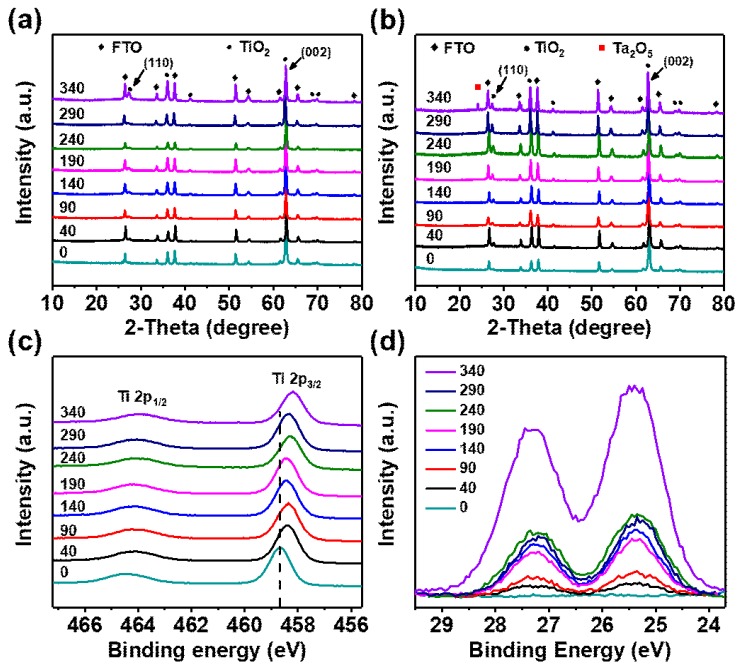
XRD patterns of Ta:TiO_2_-*v* materials (**a**) before and (**b**) after calcination. High-resolution X-ray photoelectron spectroscopy (XPS) spectra of (**c**) Ti and (**d**) Ta for Ta:TiO_2_-*v* materials.

The crystal structures of Ta:TiO_2_-*v* were examined by XRD (Figure 2a). The XRD patterns of all the Ta:TiO_2_-*v* materials in Figure 2a show three main diffraction peaks at 27.5, 36.1, and 62.8°, corresponding to the (110), (101), and (002) planes of rutile TiO_2_ (#JCPDS 21-1276) [14], respectively. The relatively higher intensity of (002) planes compared with other rutile peaks verifies the TiO_2_ nanorods grew in the [001] direction and were highly oriented with respect to the substrate surface [15]. Besides, the diffraction peaks of the FTO substrate were also detected and co-existed with Ta-doped rutile TiO_2_. However, the peak at 24.2° of Ta:TiO_2_-340 appeared after calcination (Figure 2b), illustrating the existence of Ta_2_O_5_ due to the excessive TIO. XPS spectra were used to analyze the composition and element states of all the Ta-doped TiO_2_ materials. The binding energies were calibrated according to the C 1s peak at 284.8 eV. The typical survey spectra (Figure S3) show four main photoelectron peaks at 458.3, 529.5, 27.3, and 284.8 eV, related with Ti 2p_3/2_, O 1s, Ta 4f, and C 1s, respectively. The deconvolution of high resolution XPS spectra of Ti 2p, O1s, and Ta 4f are shown in Figure S3 [16]. The presence of the C peak might come from the adsorbed CO_2_ species. Figure 2c exhibits the high-resolution spectra of Ti 2p_3/2_, indicating that the binding energies of Ti 2p peaks for all Ta-doped TiO_2_ materials were lower than that of the pristine TiO_2_. The reason for this is that the presence of Ta^5+^ may bring charge imbalance in the system, which should be equilibrated by the transition of a certain amount of Ti^4+^ to Ti^3+^. However, it is well known that Ta_2_O_5_ cannot shift the Ti 2p peak, suggesting the introduction of Ta^5+^ into the TiO_2_ lattice [11,16]. Moreover, a 4f_7/2_ peak of Ta was observed at 25.5 eV, revealing the presence of Ta^5+^ [17]. As expected, the intensity of the Ta peak increased with the increasing amount of TIO.

**Figure 3:**
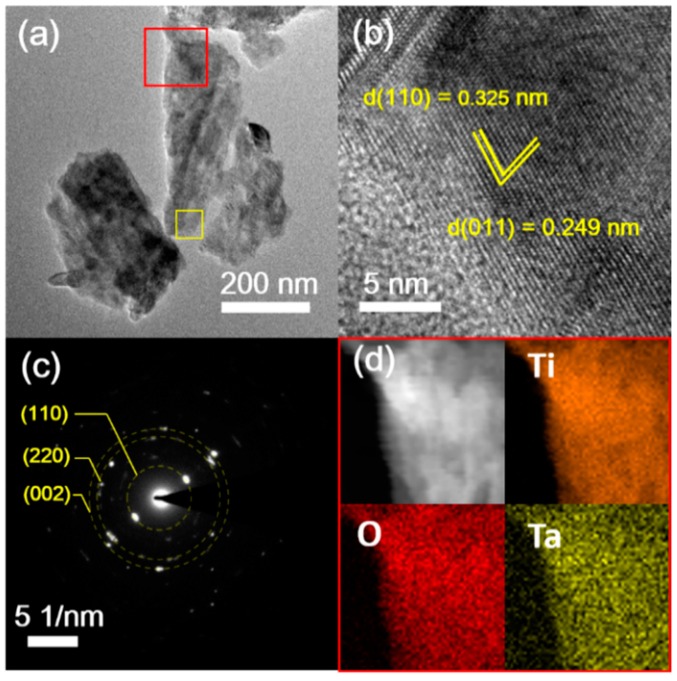
(**a**) Transmission electron microscopy (TEM), (**b)** high resolution transmission electron microscope (HRTEM), and (**c**) selected area electron diffraction (SAED) images of Ta:TiO_2_-140. (**d**) Energy-dispersive X-ray spectroscopy (EDX) mapping data of Ti (orange), O (red), and Ta (yellow).

Structural characterization of Ta:TiO_2_-140 was performed by TEM and high resolution transmission electron microscope (HRTEM). The TEM images of nanorods are shown in Figure S4, which further clearly verify the diameter of TiO_2_ nanorods at about 150 nm. Figure 3a displays the low magnification TEM image of the Ta-doped TiO_2_ particles cracked from the nanorods after ultrasonic dispersion. The HRTEM image (Figure 3b), measured with the yellow square in Figure 3a, gives a lattice fringe of about 0.325 and 0.249 nm, in agreement with the crystallographic (110) and (101) spacing of rutile TiO_2_, respectively [18]. The selected area electron diffraction (SAED) spots of the nanorod (Figure 3c) could be indexed to the (110), (220), and (002) planes, revealing the polycrystalline nature of rutile TiO_2_ [19]. Figure 3d demonstrates that Ti, Ta, and O (red square of the nanorod in Figure 3a) were homogeneously distributed through the whole area, whereas no tantalum oxide was detected by XRD or XPS measurements, which further suggests Ta elements have been successfully incorporated into TiO_2_ lattices.

### 3.2. Photoelectrochemical Performance

**Scheme 1:**
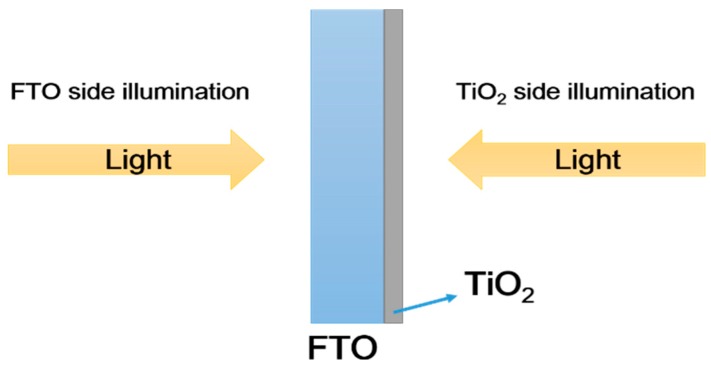
Schematic drawing of Ta:TiO_2_-*v* electrode illumination from the F-doped tin oxide (FTO) glass side (FS) and TiO_2_ side (TS).

**Figure 4:**
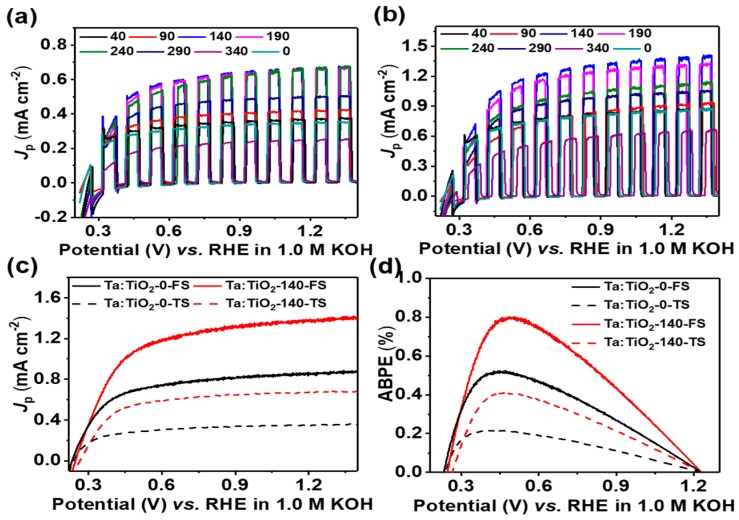
Average chopped linear sweep voltammetry (LSV) curves of Ta:TiO_2_-*v* under (**a**) TS illumination and (**b**) FS illumination. (**c**) LSV and (**d**) applied bias photon-to-current efficiency (ABPE) curves of Ta:TiO_2_-0 and -140 under TS and FS illumination.

In order to investigate the optimal Ta doping concentration, photoelectrochemical (PEC) properties of Ta:TiO_2_-*v* materials were systemically examined in 1.0 M KOH (pH = 13.6) under simulated sun light. Typically, PEC measurements were conducted employing light incident upon both the FTO glass side (FS) and TiO_2_ side (TS) (see Scheme 1). Figure 4a,b and Figure S5 show the PEC performance of all the materials under both TS and FS illumination. With increasing Ta content, the photocurrent density of Ta:TiO_2_-*v* firstly increased and then decreased in both cases, giving TiO_2_-140 (atomic ratio of Ta to Ti: 0.76 atomic %) the best performance with photocurrent densities of 0.67 and 1.36 mA cm^−2^ under TS and FS illumination, respectively. Table S2 shows that the performance of our material is comparable with that obtained with state-of-the-art TiO_2_-based photoanodes under TS illumination, and various kinds of photoanodes under FS illumination [11,17,20,21,22,23,24,25,26,27]. However, the photocurrent density decreased drastically when the TIO volume was increased above 290 μL, which may be due to the agglomerated TiO_2_ nanoparticles on the nanorods’ surface. Specifically, the performance of Ta:TiO_2_-340 was even worse than that of pristine TiO_2_, because of the surface tantalum oxide generated by the excessive TIO. Figure 4c demonstrates that the improvement in photocurrent under FS illumination can reach up to twice that under TS illumination for Ta:TiO_2_-140. According to a previous report, the enhancement of FS illumination was caused by the films’ thickness [28]. Therefore, we changed the amount of tetrabutyl titanate in the hydrothermal step to tune the length (thickness) of TiO_2_ nanorods. The cross-view of SEM images and the corresponding LSV curves are shown in Figures S6 and S7, indicating that the photocurrent difference between FS-illumination and TS-illumination became smaller with the thickness decrease. Nonetheless, Ta:TiO_2_-140 (3 μm) exhibited the highest photocurrent under FS illumination for all series materials with different thicknesses.

**Figure 5:**
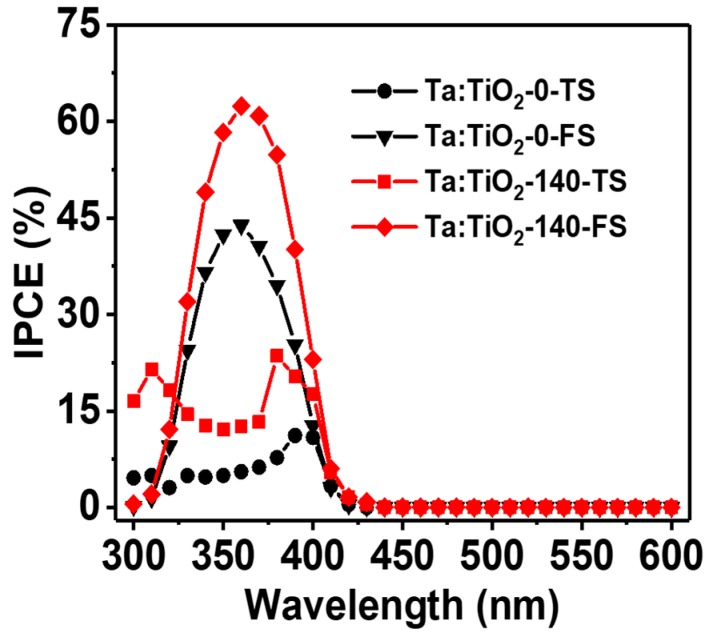
Incident-photon-to-current conversion efficiency (IPCE) of Ta:TiO_2_-0 and -140 under TS and FS illumination.

It is highly desirable to get a higher photocurrent in a relative lower bias region, since this can reduce the external power needed to drive water splitting, and thus increase the overall efficiency of PEC hydrogen generation. The applied bias photon-to-current efficiencies (ABPE, %) of Ta:TiO_2_-*v* photoanodes were calculated from the LSV curves in Figure 4c using the following Equation [29]:

ABPE = *I*(1.23 − *E*)/*J*_Light_, (1)

where *I* is the photocurrent density at the measured bias, *E* refers to the applied voltage vs. RHE, and *J*_Light_ is the irradiance intensity of 100 mW cm^−2^ (AM 1.5 G). Figure 4d presents the plots of the ABPE as a function of the applied bias. Ta:TiO_2_-140 exhibits the highest conversion efficiency of 0.51% under TS illumination and 0.80 % under FS illumination at 0.46 V, which is 2.4 and 1.5 times larger than that of Ta:TiO_2_-0, respectively. The steady-state photocurrent of Ta:TiO_2_-140 was tested at 1.23 V vs. RHE in 1.0 M KOH. It was found that the steady photocurrent densities of FS and TS illumination at 1.23 V were close to the photocurrent density in the LSV curves at the same potential (Figure 4c). Moreover, O_2_ bubbles were generated on the surface of Ta:TiO_2_-140 under FS illumination throughout the entire examination (Supplementary Video 1), and there was only 2% and 5% current loss for Ta:TiO_2_-140 under TS and FS illumination after 3 h continuous reaction, respectively (Figure S8). The stability test was suggestive of the excellent long-term stability and solar conversion application of our photoanodes. To quantitatively investigate the PEC activity, we collected incident-photon-to-current conversion efficiency (IPCE) spectra for Ta:TiO_2_-0 and -140 photoanodes at 1.23 V. The IPCE values at the specific wavelengths were calculated according the following equation: IPCE = (1240 *I*)/(λ*·P*_Light_), where *I* is the measured photocurrent density (mA cm^−2^), *λ* is the wavelength of the incident light (nm), and *P*_Light_ is the incident light irradiance (mW cm^−2^) [30]. As shown in Figure 5, the maximum IPCE value of Ta:TiO_2_-140 was around 62.4% under FS illumination at 360 nm, which was about 1.4 times higher than that of Ta:TiO_2_-0. The optimized Ta doping concentration of the TiO_2_ could improve the efficiency of the photoelectrodes. The IPCEs of Ta:TiO_2_-140 under FS illumination were enhanced significantly compared with the value achieved under TS illumination at the wavelength of 340–400 nm. However, the relatively lower efficiencies at λ < 330 nm under FS illumination were because of the glass absorption in this wavelength range.

### 3.3. Charge Separation and Injection Mechanism

To further investigate the reason for the photocurrent enhancement of Ta:TiO_2_-140 under FS illumination, three fundamental processes were systemically studied using the following Equation [31]:

*J*_Wat_ = *J*_Abs_ × *η*_Injection_ × *η*_Separation_ (2)

where *J*_Wat_ is the measured photocurrent density; *J*_Abs_ refers to the maximum photocurrent density electron flux achievable at the photoanodes; *η*_Injection_ is the efficiency of photogenerated holes injected from the electrode into the electrolyte for the desired electrochemical reaction, which is also called charge transfer efficiency in some references [31,32]; and *η*_Separation_ is the photogenerated electron-hole separation efficiency within the material [33]. The light-absorption properties of Ta:TiO_2_-*v* materials were acquired in a wavelength range from 300 to 600 nm (Figure S9), and there was no appreciable difference in light absorption despite the different Ta doping concentrations. The estimated optical bandgaps (*E*_g_) of the materials were extracted from the Tauc plots for direct bandgap transitions (Figure S9b), and the obtained values for all TiO_2_ materials were approximately 3.0 eV [34]. Hence, we obtained *J*_Abs_ by integrating the absorbance across the AM 1.5 G solar spectrum from 300 to 420 nm (Figures S10 and S11, Table S3). The approximate *J*_Abs_ values indicated that the concentration of Ta doping in our case had no obvious impact on the light absorption.

**Figure 6:**
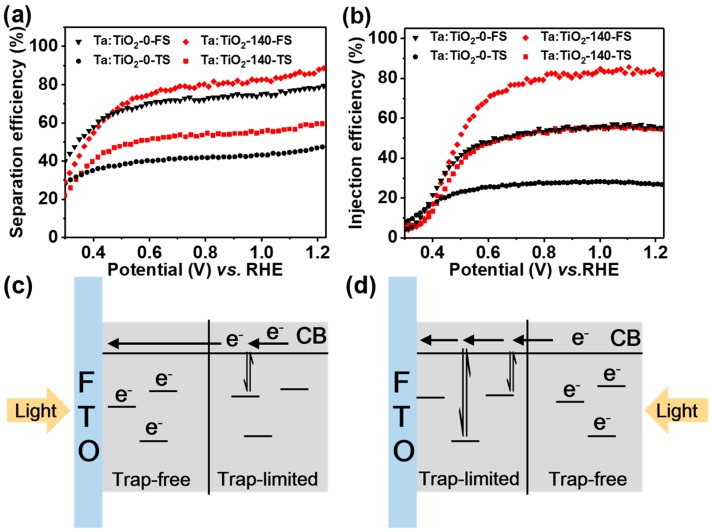
(**a**) Separation and (**b**) injection efficiencies of Ta:TiO_2_-0 and -140 under TS and FS illumination. (**c**,**d**) Schematic illustrating the electron transport behaviors.

The separation and injection efficiencies of these photoanodes were obtained via hole-scavenger-assisted PEC measurements (Figure 6a,b). Typically, the photocurrent densities of sulfite oxidation (*J*_Sul_) and water oxidation (*J*_Wat_) were conducted in 0.5 M potassium phosphate (KH_2_PO_4_) buffer solution (pH = 7.0) with and without 1.0 M Na_2_SO_3_ solution, respectively, under FS or TS illumination (Figure S12). *η*_Separation_ can be determined by the ratio of the photocurrent density of sulfite oxidation to the theoretical maximum photocurrent density, and the results are shown in Figure 6a. It is clearly seen that the separation efficiencies of Ta:TiO_2_-140 were higher than those of Ta:TiO_2_-0 under both FS and TS illumination. Ta:TiO_2_-140 under FS illumination had the highest separation efficiency of 89 % at 1.23 V vs. RHE, which was 1.1 times and 1.5 times higher than those of Ta:TiO_2_-0 under FS illumination and Ta:TiO_2_-140 under TS illumination, respectively. This can be attributed to the reducing electro-hole recombination in the electrode, which was mostly affected by the conductivity of photoanode. The PEC performance difference of Ta:TiO_2_-140 and Ta:TiO_2_-0 suggested Ta doping had an important role in decreasing the resistance of TiO_2_, which can be beneficial for increasing the separation efficiency. To explain the improvement in separation efficiency between the electrodes measured under FS and TS illumination, trap-free and trap-limited electron transport models reported previously were used [28]. Specifically, regarding FS illumination, the photogenerated electrons will fill most of the trap states to establish a trap-free transport model, since the electrons are generated near the FTO glass side. On the contrary, if the materials are measured under TS illumination, the electrons are generated in the region far from FTO, and most of the electrons should transport a mass of unfilled trap states to FTO. In this case, the trap-limited transport model will form in the zone near FTO, giving rise to a low-speed electron trapping–detrapping process. Obviously, higher electron transport speed decreases the recombination ratio in the electrode, and therefore enhances the separation efficiency.

In addition, the charge injection efficiency was calculated by dividing the photocurrent density of water oxidation into that of sulfite oxidation (*η*_Injection_ = *J*_Wat_/*J*_Sul_), and the results are shown in Figure 6b [35]. The key assumption for this approach is that the surface recombination is negligible, since the consumption of the surface-reaching hole is considered to be 100% using the scavenger (Na_2_SO_3_) [33]. Ta:TiO_2_-140 still exhibited notably higher injection efficiency than that of Ta:TiO_2_-0, suggesting the presence of Ta effectively reduced the surface recombination of charge carriers. Moreover, the surface nanoparticles can dramatically increase the contact area between electrode and electrolyte, thus increasing the injection efficiency. On the other hand, the photoelectrodes measured under FS illumination also showed higher injection efficiency than under TS illumination. The generated electrons can be removed quickly across the trap-free states region of the electrode under FS illumination. Hence, a lower electron accumulation means a lower electron-hole recombination on the surface, and thus a higher injection efficiency of the electrode.

### 3.4. EIS and Band Structure Characteristics

**Figure 7:**
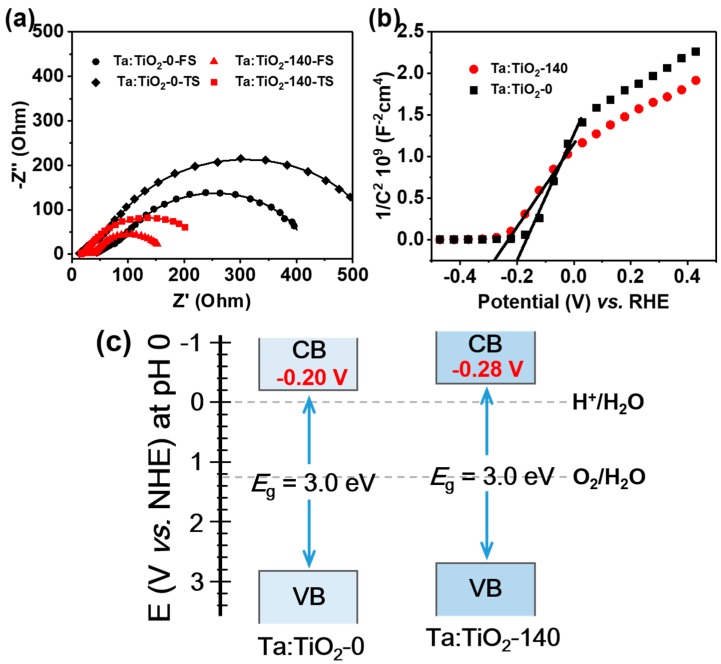
(**a**) Electrochemical impedance spectroscopy of Ta:TiO_2_-0 and -140 under TS and FS illumination. (**b**) Mott–Schottky plots and (**c**) band structure diagram of Ta:TiO_2_-0 and -140.

Electrochemical impedance spectroscopy (EIS) curves were measured under the open-circuit condition in the frequency range of 10^4^–0.1 Hz. It is well known that the semicircle at the high frequency zone represents electron transport inside the bulk (*R*_CT1_), and the low frequency semicircle is equal to the electrode–electrolyte interface resistance (*R*_CT2_) [36]. The diameter of the semicircle in the Nyquist plots reflects the charge transfer resistance, and the large value of impedance elucidates the poor electron conductivity of the photoelectrode [37]. Therefore, the measured EIS raw data (Figure 7a and Figure S13) should be fitted into an equivalent circuit (Figure S14) using Zsimpwin software (the fitted results are shown in Tables S4 and S5). As shown in Figure 7a, the relatively lower resistance of electron transport inside the electrode (*R*_CT1_) for the materials using intermediate volume of Ta precursor, especially for Ta:TiO_2_-140, indicates the proper Ta doping concentration can accelerate the electron transfer during the reaction. In addition, the low electrode–electrolyte interface resistance (*R*_CT2_) of Ta:TiO_2_-140 was due to the high contact area of abundant nanoparticles on the top surface, which can promote surface water oxidation [38]. The materials under FS illumination exhibited smaller semicircles than those under TS illumination, implying the trap-free states significantly facilitate the electron transportation inside the electrode, and thus boost the surface water oxidation [39]. These results agree well with the separation and injection efficiencies analysis, as we discussed above.

The Mott–Schottky (M–S) plots were conducted at a fixed frequency (1 KHz). As shown in Figure 7b, the slopes of the linear parts of the curves in the M–S plots were positive, indicating the materials are *n*-type semiconductors. The donor densities (*N*_D_) of Ta:TiO_2_-*v* were acquired from the following Equation [40]:

*N*_D_ = (2/*eεε*_0_)/[d(1/*C*^2^)/d*V*] (3)

where *C* is the space charge layers capacitance, *e* is the electron charge, *ε* is the dielectric constant, and *ε*_0_ is the permittivity of a vacuum. The *N*_D_ of Ta:TiO_2_-0 and Ta:TiO_2_-140 were calculated to be 9.22 × 10^19^ and 1.69 × 10^20^ cm^−3^, respectively. The donor density significantly increases after Ta doping. Therefore, the increased donor density is expected to shift the Fermi level of TiO_2_ toward the conduction band, which further facilitates the charge separation at the semiconductor–electrolyte interface, and improves its PEC performance [41]. Besides, the flat band potential (*E*_FB_) can be calculated through the intercept of the tangent line in the M–S plots, and the values were determined to be −0.20 and −0.28 V (vs. normal hydrogen electrode, NHE, pH = 0) for Ta:TiO_2_-0 and Ta:TiO_2_-140, respectively. The gap between the flat band potential and the bottom edge of the conduction band is negligible for *n*-type semiconductors [42]. Thus, the flat band potential is approximately equal to the conduction band. In our case, no evidence was observed to show a band gap change after Ta doping from the UV-Vis absorption spectra and its corresponding Tauc plot results (Figure S8). Therefore, there must have been some shift of the conduction band edge to complement the valence band edge change, which has been further confirmed by the valence band XPS spectra of Ta:TiO_2_-0 and -140 (Figure S15) [9,13,43,44]. The potential energy diagram in Figure 7c illustrates that the conduction band and valence band showed a shift in the cathodic direction by 0.08 V after Ta doping. The negative shift of the conduction band is beneficial for hydrogen production and charge recombination decrease, thus improving the PEC performance [45].

## 4. Conclusions

In summary, we have developed hierarchical Ta doped TiO_2_ nanorod arrays, with nanoparticles on the top, on FTO for efficient PEC electrodes. The photoelectrocatalytic activity enhancement of Ta:TiO_2_ can be attributed to the following three parameters: Ta doping, surface nanoparticles, and the FS illumination model. Firstly, Ta doping can reduce both bulk and surface electron-hole recombination by increasing the electron conductivity, as well as upward shift the flat potential to a negative potential. Secondly, the surface nanoparticles on the top of Ta:TiO_2_ nanorods effectively increase the interface area between the electrolyte and the active material, thus promoting charge injection and reducing electrode–electrolyte interface resistance. Finally, the electrons have higher transport speed through the trap-free region near the FTO glass under FS illumination, giving higher charge separation efficiency and lower electron transport resistance. Therefore, our work gives a remarkable strategy for designing and constructing low-cost and effective photoanodes for practical sunlight-driven photoelectrochemical water-splitting.

## Supplementary Materials

The following are available online at http://www.mdpi.com/2079-4991/8/12/983/s1. Figure S1: Surface SEM images of Ta:TiO_2_-*v*, Figure S2: Cross SEM images of Ta:TiO_2_-*v*, Figure S3: (a) XPS survey of Ta:TiO_2_-*v*. The deconvolution of high resolution XPS spectra for (b) O1s, (c) Ti 2P, and (d) Ta 4f of Ta:TiO_2_-*v*, Figure S4: TEM images of Ta:TiO_2_-140 nanorods, Figure S5: (a,b) ABPE and (c,d) chopped photocurrent density vs. time of Ta:TiO_2_-*v* under TS illumination and FS illumination, Figure S6: Cross SEM images of Ta doped TiO_2_ with different thicknesses (different volume of tetrabutyl titanate), Figure S7: (a) LSV curves, (b) current densities at 1.23 V vs. RHE, and (c) the ratio of *J*_FS_ to *J*_TS_ for Ta doped TiO_2_ with different thicknesses, Figure S8: Chronoamperometry curves of water oxidation over Ta:TiO_2_-140 photoanode at 1.23 V (vs. RHE), Figure S9: (a) UV-Vis absorption spectra and (b) Tauc plots of Ta:TiO_2_-*v*. Figure S10: (a) Diffuse reflectance spectra, (b) transmission spectra, (c) UV-Vis absorption properties (*η*_abs_), and (d) the light harvesting efficiencies (*η*_LHE_) of Ta:TiO_2_-*v*, Figure S11: (a) Electron flux of AM 1.5 G solar spectrum. (b) Electro flux of Ta:TiO_2_-*v*, Figure S12: LSV curves of Ta:TiO_2_-0 and -140 for water oxidation (WO, blue line) measured in 0.5 M phosphate buffer (pH = 7), and sulfite oxidation (SO, red line) measured in 0.5 M phosphate buffer in the presence of 1 M Na_2_SO_3_ (pH = 7), under TS and FS illumination. The black line was measured in 0.5 M phosphate buffer in the presence of 1 M Na_2_SO_3_ without illumination, Figure S13: Nyquist plots and magnified Nyquist plots of Ta:TiO_2_-*v* under (a,b) TS illumination and (c,d) FS illumination. The experimental data and simulated impedance response are represented by discrete points and solid lines, respectively, Figure S14: Equivalent circuit used for EIS data fitting. *R*_s_ refers to the solution resistance, *R*_CT1_ is the charge transfer resistance within the electrode, *R*_CT2_ represents the resistance to electron transport at the electrode–electrolyte interface, *CPE* is the constant phase angle element, Figure S15: Valence band XPS spectra of Ta:TiO_2_-0 and -140, Table S1: SEM-based EDX results for Ta/Ti atomic percentage (%) of Ta:TiO_2_-*v*, Table S2: A summary of recent results of the photocatalytic performance of various photoanodes in photoelectrochemical water oxidation, Table S3: Maximum achievable photocurrent density electron flux of Ta:TiO_2_-*v*, Table S4: The values of *R* and *CPE* derived by fitting the EIS of Ta:TiO_2_-*v* under TS illumination, Table S5: The values of *R* and *CPE* derived by fitting the EIS of Ta:TiO_2_-*v* under FS illumination*,* Video S1: Photoelectrochemical testing of Ta:TiO_2_-140.
