# Supplementary material for: Hierarchical Ta-Doped TiO2 Nanorod Arrays with Improved Charge Separation for Photoelectrochemical Water Oxidation under FTO Side Illumination
Source: Nanomaterials (Basel). 2018 Nov 28;8(12):983. doi: 10.3390/nano8120983 (PMC6316417; doi:10.3390/nano8120983)
Supplement: Supplementary file 1 [file nanomaterials-08-00983-s001.zip › nanomaterials-382548-SI/nanomaterials-382548-for published-supplementary.docx]

**Supporting Information (SI) for:**

**Hierarchical Ta-Doped TiO_2_ Nanorod Arrays with Improved Charge Separation for Photoelectrochemical Water Oxidation under FTO Side Illumination**


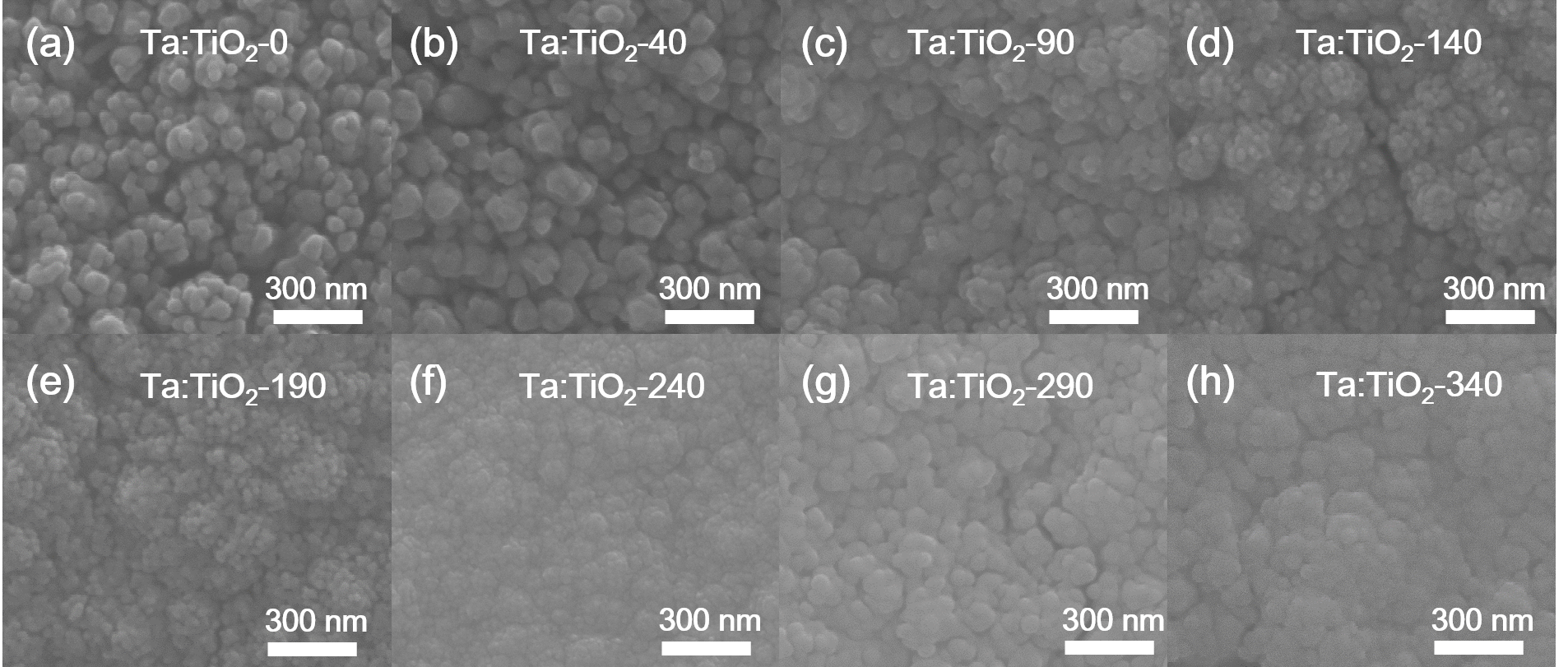


**Figure S1.** Surface SEM images of Ta:TiO_2_-*v*.


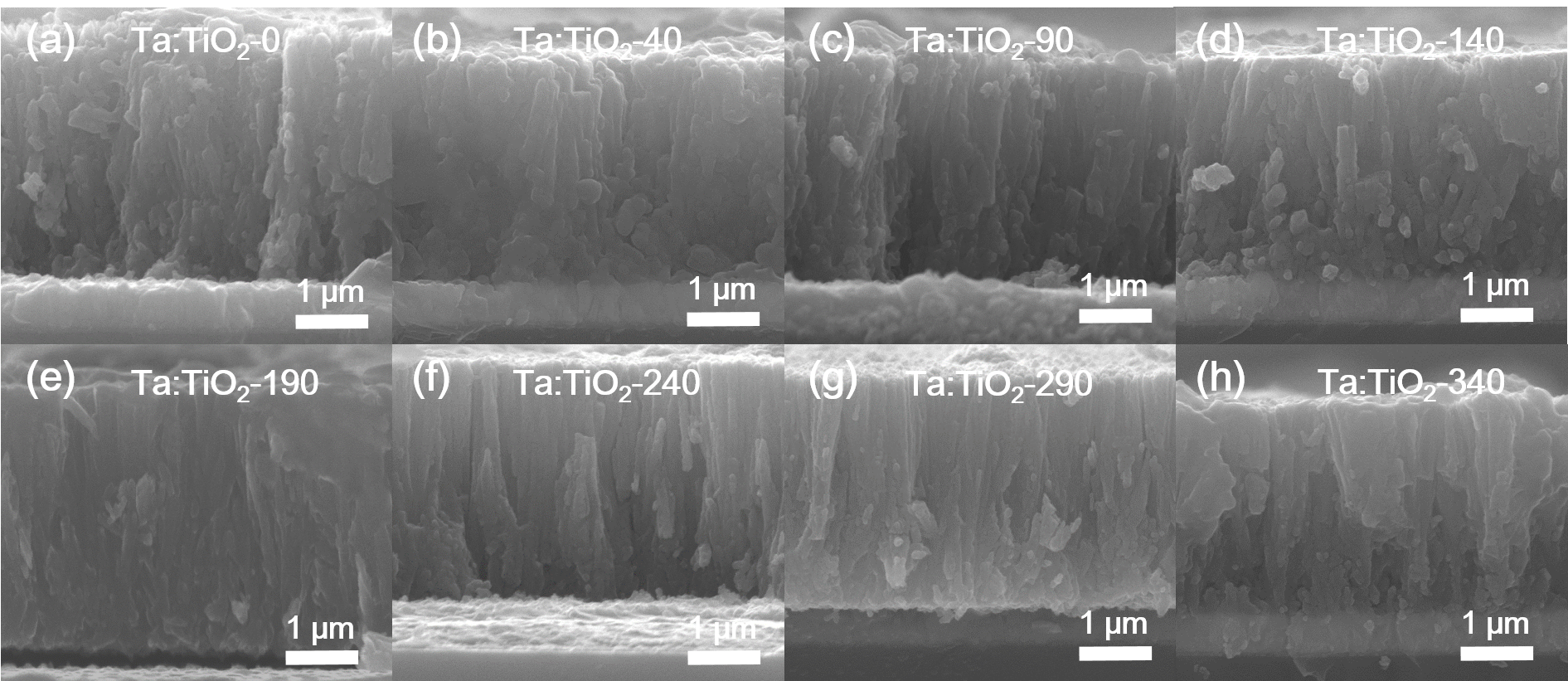


**Figure S2.** Cross SEM images of Ta:TiO_2_-*v*.

**Table S1.** SEM-based EDX results for Ta/Ti atomic percentage (%) of Ta:TiO_2_-*v*.

| **Samples** | **1#** | **2#** | **3#** | **4#** | **5#** | **Mean** |
| --- | --- | --- | --- | --- | --- | --- |
| Ta:TiO_2_-40 (%) | 0.375 | 0.290 | 0.311 | 0.400 | 0.370 | 0.349 |
| Ta:TiO_2_-90(%) | 0.518 | 0.487 | 0.458 | 0.508 | 0.449 | 0.484 |
| Ta:TiO_2_-140(%) | 0.812 | 0.724 | 0.785 | 0.776 | 0.701 | 0.760 |
| Ta:TiO_2_-190(%) | 1.187 | 0.909 | 1.314 | 0.976 | 0.787 | 1.035 |
| Ta:TiO_2_-240(%) | 1.906 | 1.609 | 1.496 | 1.665 | 1.320 | 1.600 |
| Ta:TiO_2_-290(%) | 2.516 | 2.689 | 2.897 | 2.694 | 3.064 | 2.772 |
| Ta:TiO_2_-340(%) | 4.868 | 4.888 | 4.762 | 4.843 | 4.997 | 4.872 |


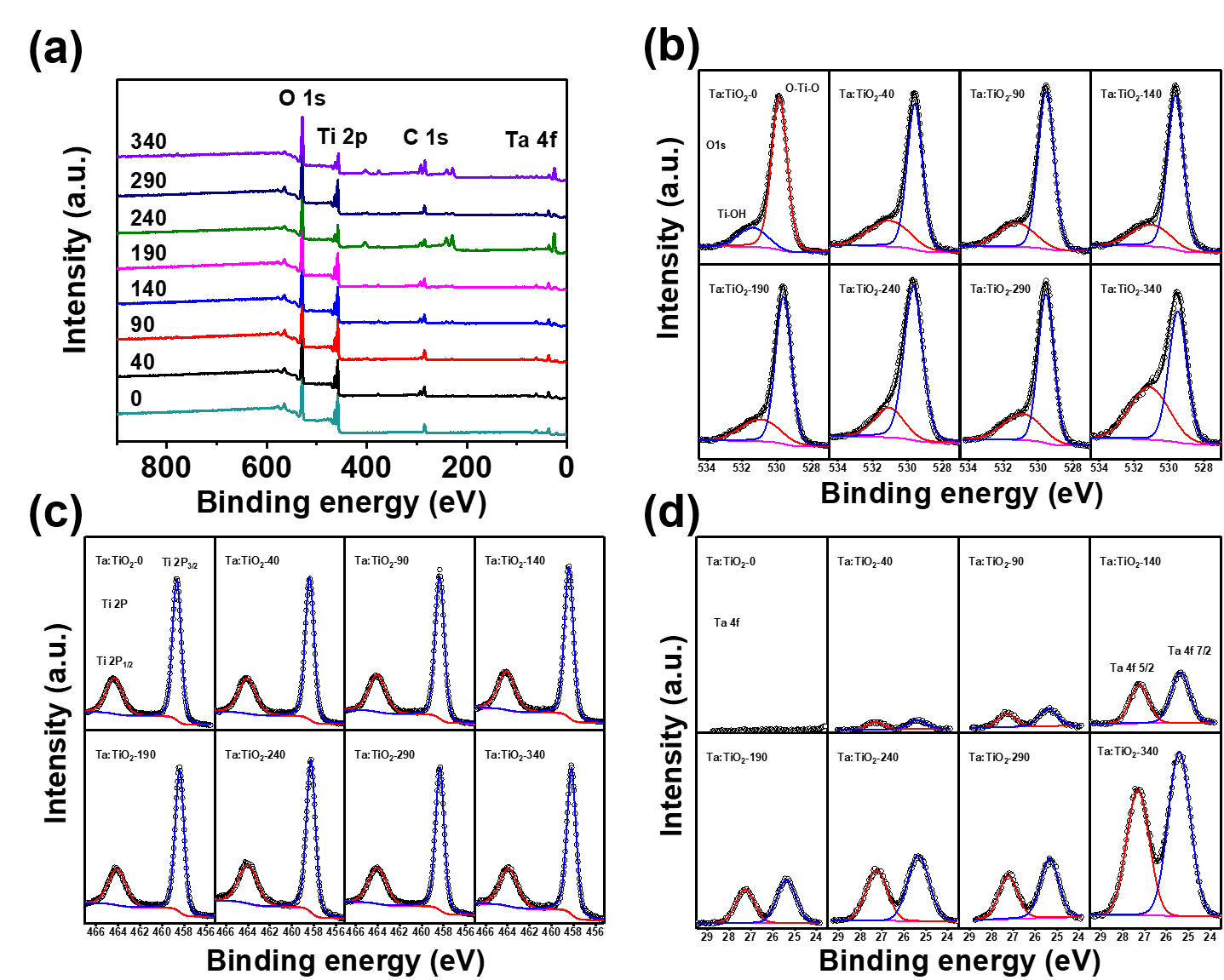


**Figure S3.** (**a**) XPS survey of Ta:TiO_2_-*v*. The deconvolution of high resolution XPS spectra for (**b**) O1s, (**c**) Ti 2P, and (**d**) Ta 4f of Ta:TiO_2_-*v*.


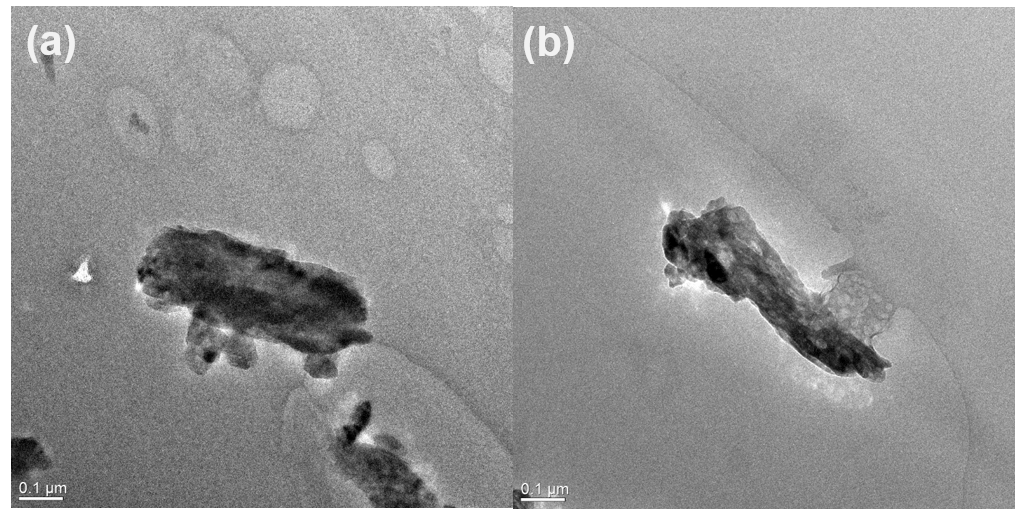


**Figure S4.** TEM images of Ta:TiO_2_-140 nanorods.


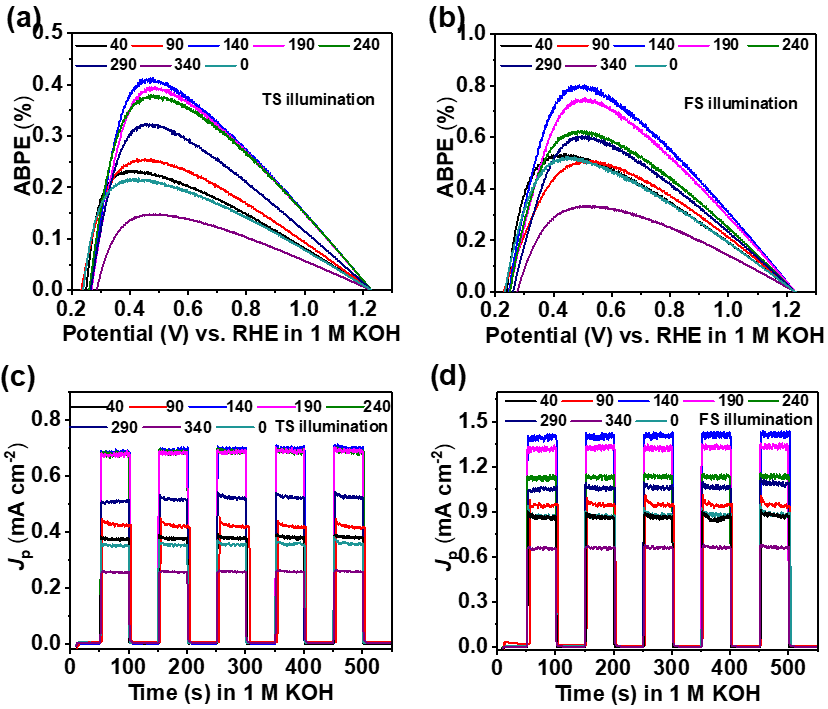


**Figure S5.** (**a,b**) ABPE and (**c,d**) chopped photocurrent density vs. time of Ta:TiO_2_-*v* under TS illumination and FS illumination.

**Table S2.** A summary of recent results of the photocatalytic performance of various photoanodes in photoelectrochemical water oxidation.

| **Materials** | **Solution** | **Light** | **Illumination model** | **Potential**  **(V)** | **Photocurrent (mA cm^−2^)** | **Reference** |
| --- | --- | --- | --- | --- | --- | --- |
| Mesoporous Ta doped TiO_2_ | 0.1 M NaOH | 200 mW cm^−2^ | Front | 1.23 | 0.25 | [1](#_ENREF_1) |
| Ta doped TiO_2_ nanotube | 1 M KOH | AM 1.5 G | Front | 1.23 | 0.53 | [2](#_ENREF_2) |
| Ta doped TiO_2_ nanotube | 0.1 M Na_2_SO_4_ | AM 1.5 G | Front | 1.23 | ~0.62 | [3](#_ENREF_3) |
| Anatase-rutile TiO_2_ | 1 M KOH | AM 1.5 G | Front | 1.23 | 0.63 | [4](#_ENREF_4) |
| Ti^3+^ doped TiO_2_ | 1 M KOH | AM 1.5 G | Front | 1.23 | 0.7 | [5](#_ENREF_5) |
| **Hierarchical Ta doped TiO_2_** | **1 M KOH** | **AM 1.5 G** | **Front** | **1.23** | **0.67** | **This Work** |
| CoPi/Fe_2_O_3_ | 1 M KPi | AM 1.5 G | Back | 1.23 | ~0.5 | [6](#_ENREF_6) |
| ZnO/BiVO_4_ | KPi | AM 1.5 G | Back | 1.23 | ~1.4 | [7](#_ENREF_7) |
| CoPi/TiO_2_ | 0.1 M KPi | AM 1.5 G | Back | 1.23 | ~0.5 | [8](#_ENREF_8) |
| Mo doped BiVO_4_ | 0.5 M Na_2_SO_4_ | AM 1.5 G | Back | 1.23 | ~0.8 | [9](#_ENREF_9) |
| WO_3_ nanocrystals | 0.5 M Na_2_SO_4_ | AM 1.5 G | Back | 1.23 | ~0.6 | [10](#_ENREF_10) |
| **Hierarchical Ta doped TiO_2_** | **1 M KOH** | **AM 1.5 G** | **Back** | **1.23** | **1.37** | **This Work** |

**
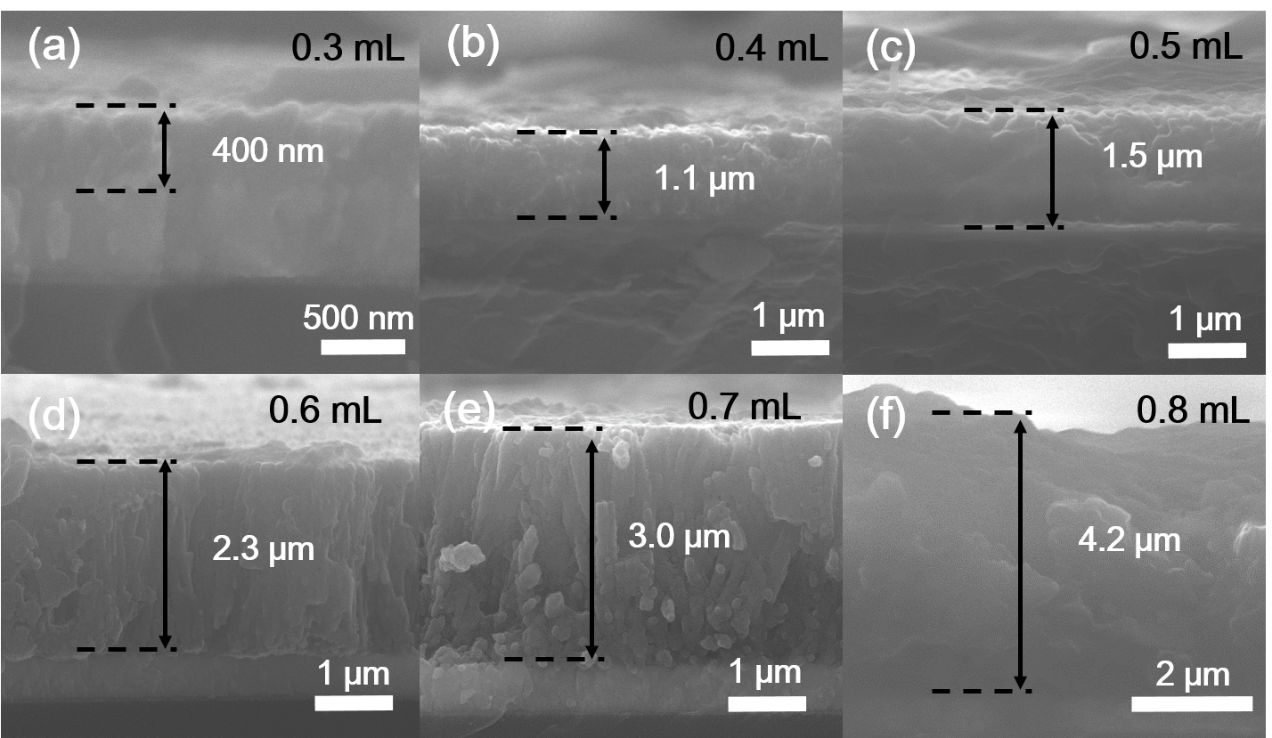
 Figure S6.** Cross SEM images of Ta doped TiO_2_ with different thicknesses (different volume of tetrabutyl titanate).


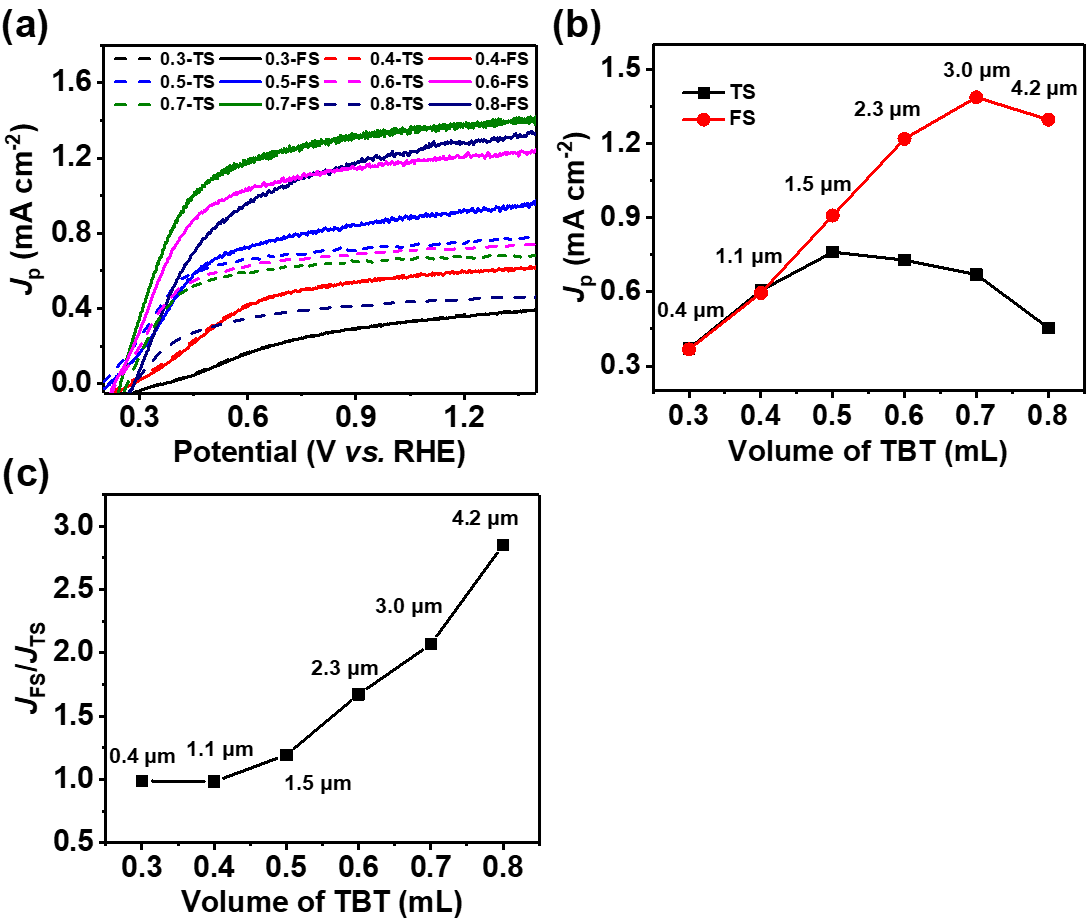


**Figure S7.** (**a**) LSV curves, (**b**) current densities at 1.23 V vs. RHE, and (**c**) the ratio of *J*_FS_ to *J*_TS_ for Ta doped TiO_2_ with different thicknesses.


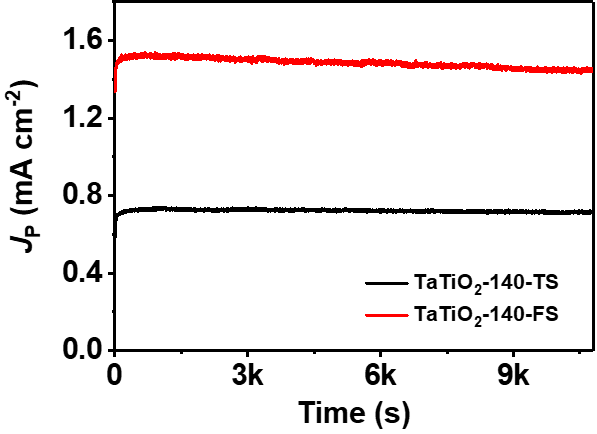


**Figure S8.** Chronoamperometry curves of water oxidation over Ta:TiO_2_-140 photoanode at 1.23 V (vs. RHE).


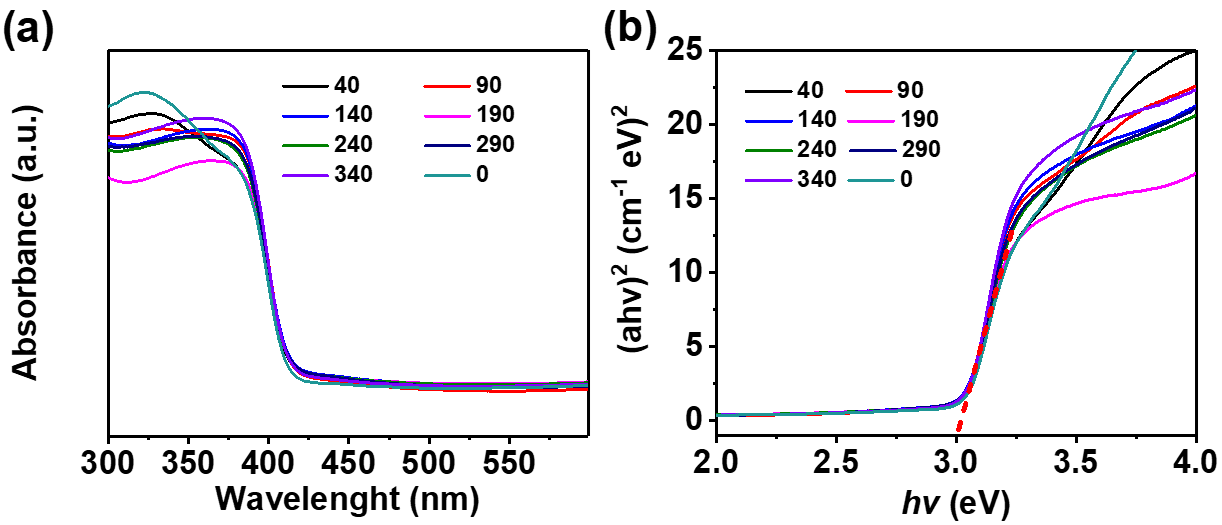


**Figure S9.** (**a**) UV-Vis absorption spectra and (**b**) Tauc plots of Ta:TiO_2_-*v*.


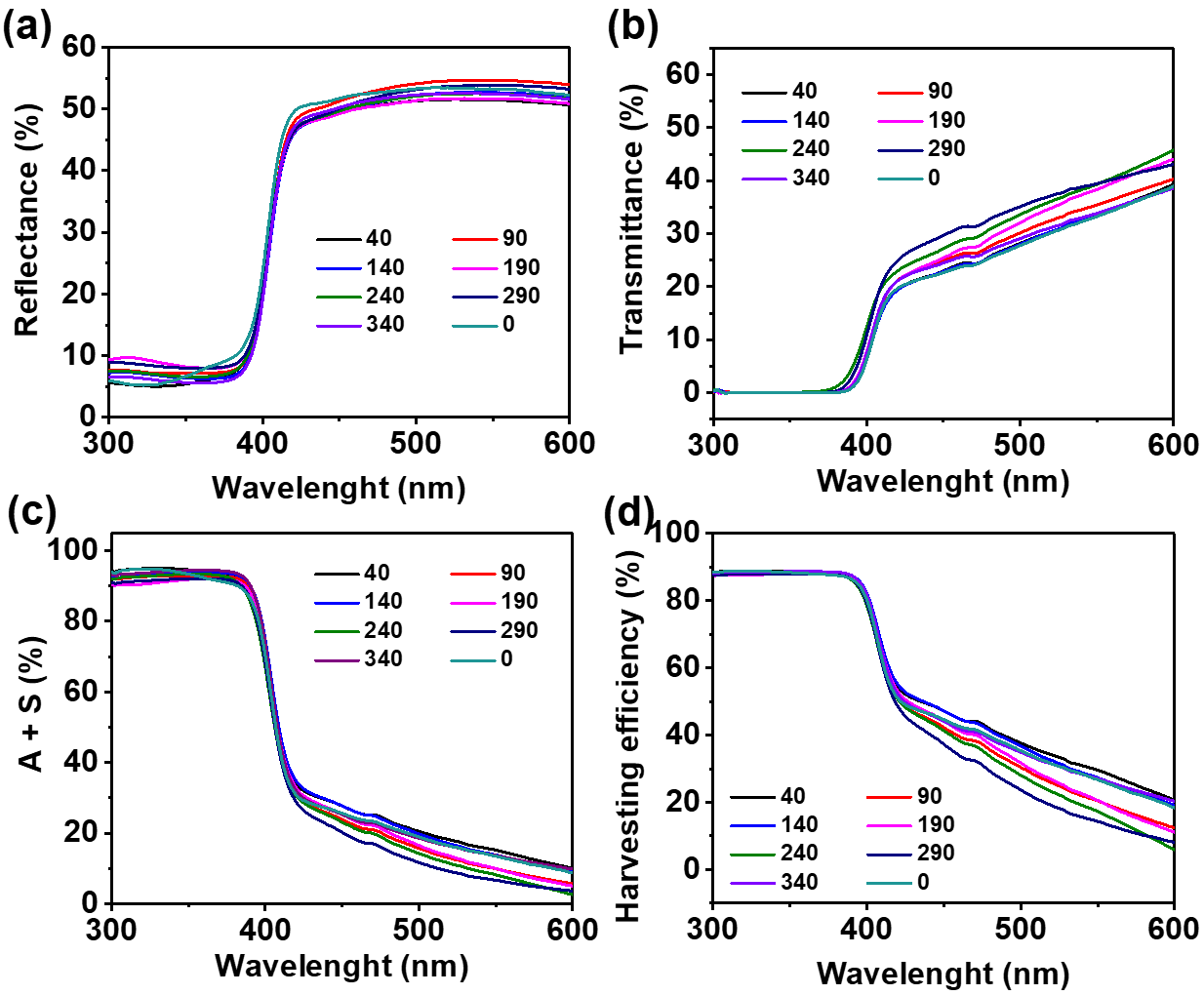


**Figure S10.** (**a**) Diffuse reflectance spectra, (**b**) transmission spectra, (**c**) UV-Vis absorption properties (*η*_abs_), and (**d**) the light harvesting efficiencies (*η*_LHE_) of Ta:TiO_2_-*v*.


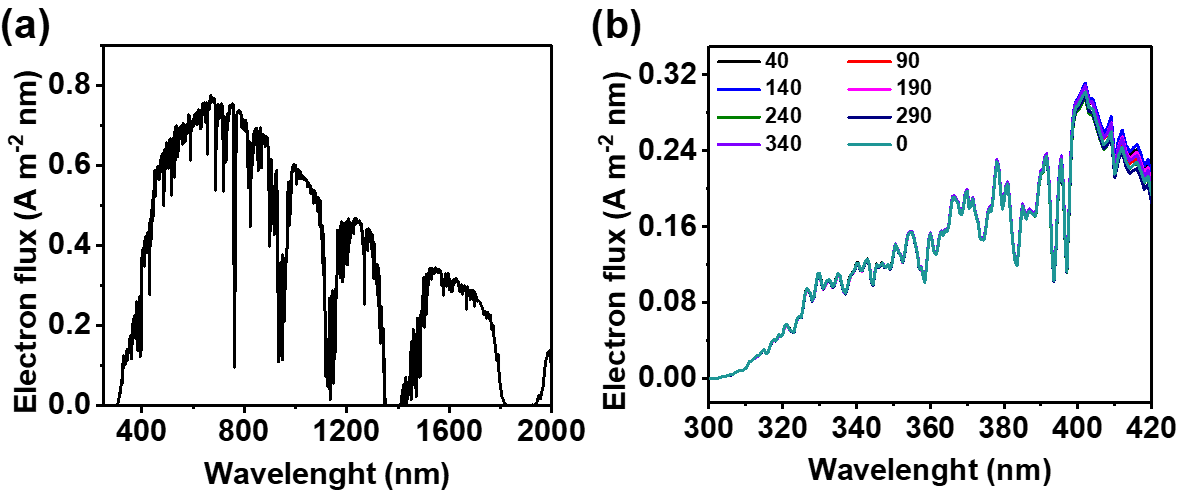


**Figure S11.** (**a**) Electron flux of AM 1.5 G solar spectrum. (**b**) Electro flux of Ta:TiO_2_-*v*.

**Table S3.** Maximum achievable photocurrent density electron flux of Ta:TiO_2_-*v*.

| **Samples** | Ta:TiO_2_-40 | Ta:TiO_2_-90 | Ta:TiO_2_-140 | Ta:TiO_2_-190 | Ta:TiO_2_-240 | Ta:TiO_2_-290 | Ta:TiO_2_-340 | Ta:TiO_2_-0 |
| --- | --- | --- | --- | --- | --- | --- | --- | --- |
| ***J*_Abs_**  **mA/cm^2^** | 1.6986 | 1.6869 | 1.7086 | 1.6886 | 1.6643 | 1.6592 | 1.6957 | 1.6727 |


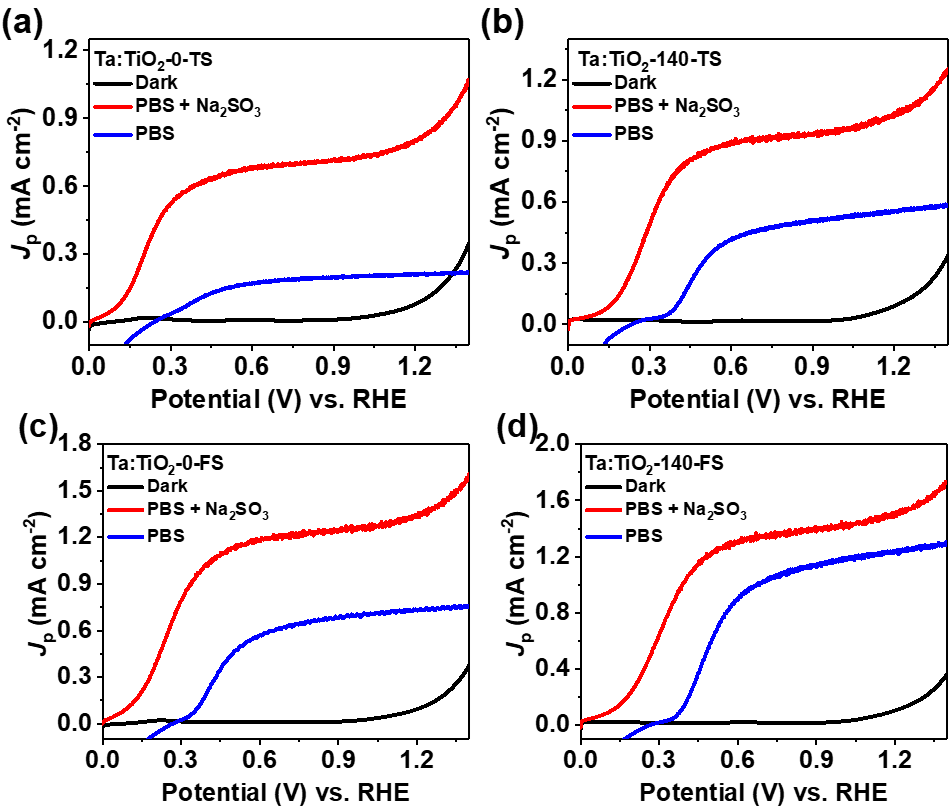


**Figure S12.** LSV curves of Ta:TiO_2_-0 and -140 for water oxidation (WO, blue line) measured in 0.5 M phosphate buffer (pH = 7), and sulfite oxidation (SO, red line) measured in 0.5 M phosphate buffer in the presence of 1 M Na_2_SO_3_ (pH = 7), under TS and FS illumination. The black line was measured in 0.5 M phosphate buffer in the presence of 1 M Na_2_SO_3_ without illumination.


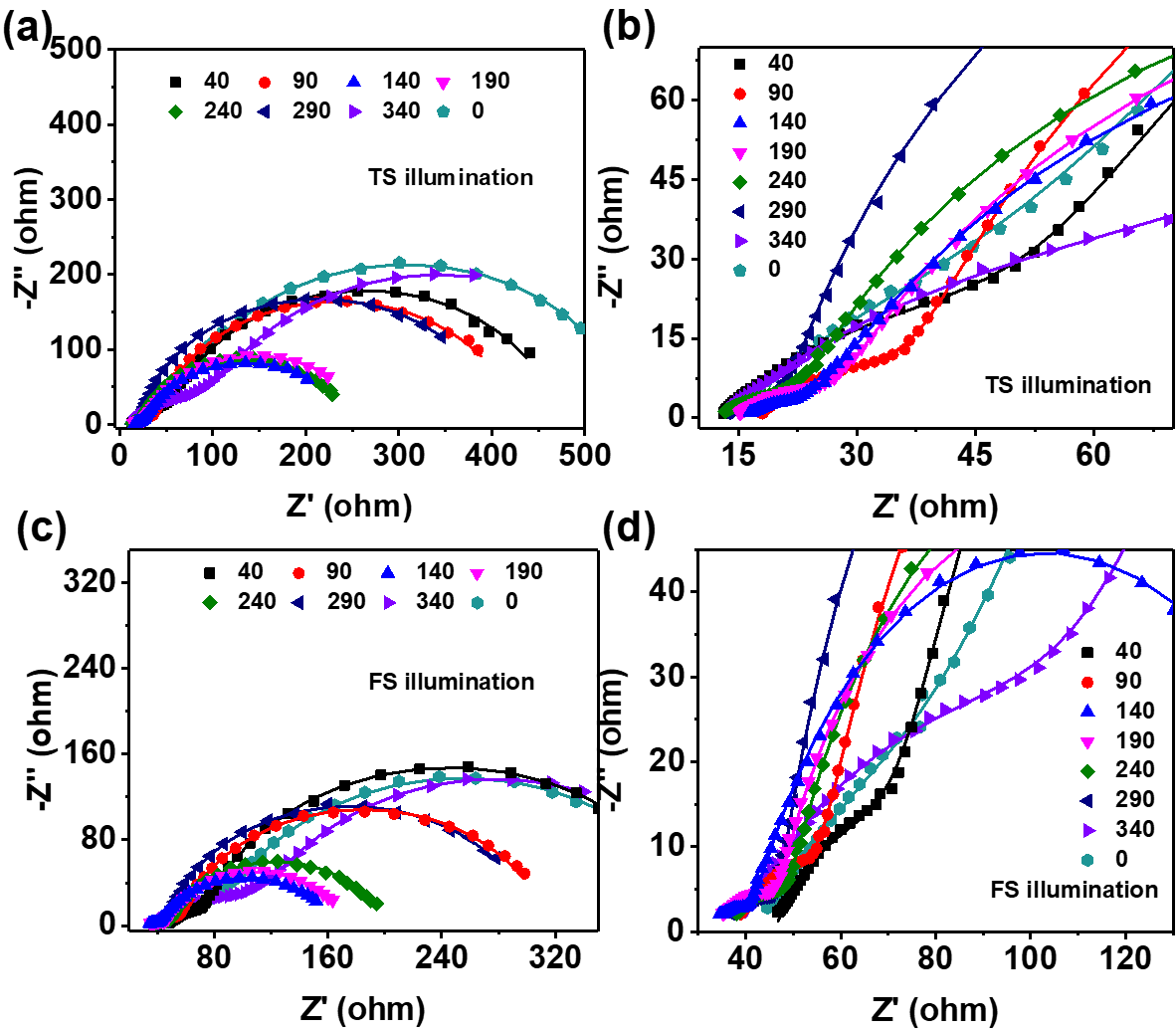


**Figure S13.** Nyquist plots and magnified Nyquist plots of Ta:TiO_2_-*v* under (**a,b**) TS illumination and (**c,d**) FS illumination. The experimental data and simulated impedance response are represented by discrete points and solid lines, respectively.


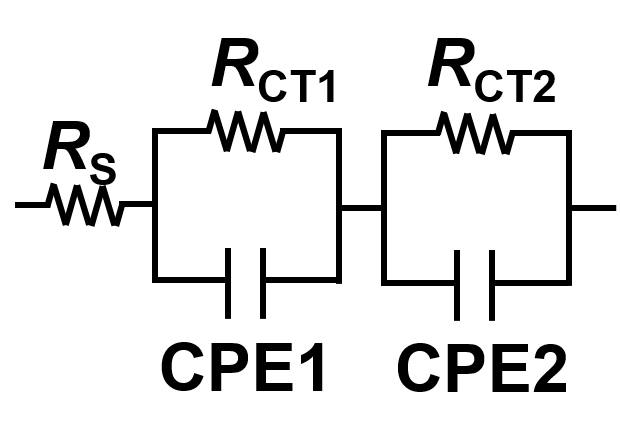


**Figure S14.** Equivalent circuit used for EIS data fitting. *R*_s_ refers to the solution resistance, *R*_CT1_ is the charge transfer resistance within the electrode, *R*_CT2_ represents the resistance to electron transport at the electrode–electrolyte interface, *CPE* is the constant phase angle element.

**Table S4.** The values of *R* and *CPE* derived by fitting the EIS of Ta:TiO_2_-*v* under TS illumination.

| **Samples (TS)** | ***R*_S_**  **(ohm)** | ***R*_CT1_**  **(ohm)** | ***CPE*1-Y_0_**  **(μF)** | ***CPE1-n*** | ***R*_CT2_**  **(ohm)** | ***CPE*2-Y_0_**  **(μF)** | ***CPE2-n*** |
| --- | --- | --- | --- | --- | --- | --- | --- |
| Ta:TiO_2_-40 | 12.82 | **48.11** | 738.9 | 0.67 | **411.6** | 437.6 | 0.90 |
| Ta:TiO_2_-90 | 17.65 | **21.01** | 436 | 0.69 | **392** | 873.6 | 0.89 |
| Ta:TiO_2_-140 | 15.16 | **13.01** | 3573 | 0.54 | **219.3** | 2238 | 0.81 |
| Ta:TiO_2_-190 | 14.56 | **13.63** | 1839 | 0.65 | **239.7** | 637.3 | 0.83 |
| Ta:TiO_2_-240 | 11.99 | **13.16** | 1206 | 0.55 | **219.5** | 1042 | 0.86 |
| Ta:TiO_2_-290 | 12.31 | **19.61** | 1355 | 0.55 | **386.5** | 1246 | 0.91 |
| Ta:TiO_2_-340 | 12.82 | **104.1** | 792.2 | 0.59 | **468.7** | 2229 | 0.88 |
| Ta:TiO_2_-0 | 12.76 | **69.82** | 966 | 0.59 | **472.5** | 635.1 | 0.92 |

**Table S5.** The values of *R* and *CPE* derived by fitting the EIS of Ta:TiO_2_-*v* under FS illumination.

| **Samples (FS)** | ***R*_S_**  **(ohm)** | ***R*_CT1_**  **(ohm)** | ***CPE*1-Y_0_**  **(μF)** | ***CPE1-n*** | ***R*_CT2_**  **(ohm)** | ***CPE*2-Y_0_**  **(μF)** | ***CPE2-n*** |
| --- | --- | --- | --- | --- | --- | --- | --- |
| Ta:TiO_2_-40 | 46.06 | **29.62** | 452.2 | 0.66 | **344** | 791.4 | 0.90 |
| Ta:TiO_2_-90 | 37.91 | **19.48** | 415.2 | 0.65 | **256.1** | 933.6 | 0.89 |
| Ta:TiO_2_-140 | 30.24 | **13.06** | 1641 | 0.41 | **120.9** | 2147 | 0.80 |
| Ta:TiO_2_-190 | 33.36 | **14.41** | 559.9 | 0.60 | **124.4** | 1839 | 0.86 |
| Ta:TiO_2_-240 | 36.38 | **13.66** | 411.2 | 0.62 | **148.2** | 1107 | 0.86 |
| Ta:TiO_2_-290 | 34.54 | **13.68** | 767.7 | 0.55 | **255.7** | 1287 | 0.91 |
| Ta:TiO_2_-340 | 38.42 | **93** | 743.5 | 0.55 | **309.6** | 2511 | 0.90 |
| Ta:TiO_2_-0 | 42.37 | **64.67** | 931.1 | 0.53 | **315.7** | 736.7 | 0.89 |


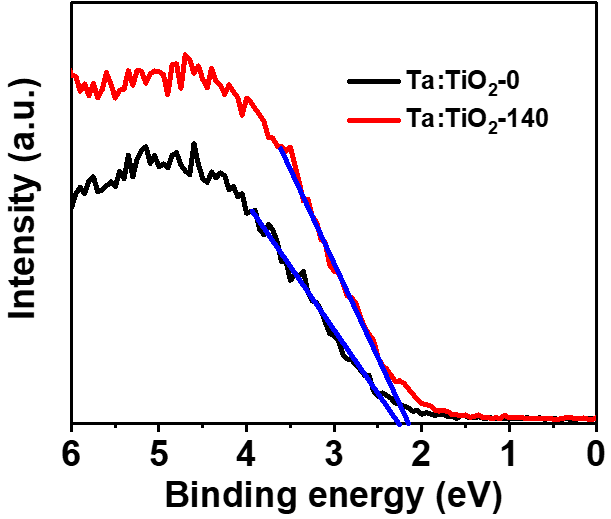


**Figure S15.** Valence band XPS spectra of Ta:TiO_2_-0 and -140.

References

1. Reitz, C.; Reinacher, J.; Hartmann, P.; Brezesinski, T. Polymer-templated ordered large-pore mesoporous anatase rutile TiO_2_:Ta nanocomposite films: Microstructure, electrical conductivity, and photocatalytic and photoelectrochemical properties. *Catal. Today* **2014**, *225*, 55–63.
2. Altomare, M.; Lee, K.; Killian, M.S.; Selli, E.; Schmuki, P. Ta-Doped TiO_2_ nanotubes for enhanced solar-light photoelectrochemical water splitting. *Chem.-Eur. J.* **2013**, *19*, 5841–5844.
3. Yan, Y.; Lee, J.; Cui, X. Enhanced photoelectrochemical properties of Ta-TiO_2_ nanotube arrays prepared by magnetron sputtering. *Vacuum* **2017**, *138*, 30–38.
4. Li, A.; Wang, Z.; Yin, H.; Wang, S.; Yan, P.; Huang, B.; Wang, X.; Li, R.; Zong, X.; Han, H.; et al. Understanding the anatase-rutile phase junction in charge separation and transfer in a TiO_2_ electrode for photoelectrochemical water splitting. *Chem. Sci.* **2016**, *7*, 6076–6082.
5. Mao, C.Y.; Zuo, F.; Hou, Y.; Bu, X.H.; Feng, P.Y. In situ preparation of a Ti^3+^ self-doped TiO_2_ film with enhanced activity as photoanode by N_2_H_4_ reduction. *Angew. Chem. Int. Ed.* **2014**, *53*, 10485–10489.
6. Carroll, G. M.; Zhong, D.K.; Gamelin, D.R., Mechanistic insights into solar water oxidation by cobalt-phosphate-modified alpha-Fe_2_O_3_ photoanodes. *Energy Environ. Sci.* **2015**, *8*, 577–584.
7. Zhang, L.W.; Reisner, E.; Baumberg, J.J. Al-doped ZnO inverse opal networks as efficient electron collectors in BiVO_4_ photoanodes for solar water oxidation. *Energy Environ. Sci.* **2014**, *7*, 1402–1408.
8. Ai, G.J.; Mo, R.; Li, H.X.; Zhong, J.X. Cobalt phosphate modified TiO_2_ nanowire arrays as co-catalysts for solar water splitting. *Nanoscale* **2015**, *7*, 6722–6728.
9. Antony, R.P.; Bassi, P.S.; Abdi, F.F.; Chiam, S.Y.; Ren, Y.; Barber, J.; Loo, J.S.C.; Wong, L.H. Electrospun Mo-BiVO_4_ for Efficient Photoelectrochemical Water Oxidation: Direct Evidence of Improved Hole Diffusion Length and Charge separation. *Electrochim. Acta* **2016**, *211*, 173–182.
10. Hong, S.J.; Jun, H.; Borse, P.H.; Lee, J.S. Size effects of WO_3_ nanocrystals for photooxidation of water in particulate suspension and photoelectrochemical film systems. *Int. J. Hydrogen Energ* **2009**–3242.
